# Making medicines more accessible in China: An empirical study investigating the early progress of essential medicine system

**DOI:** 10.1371/journal.pone.0201582

**Published:** 2018-08-02

**Authors:** Yan Song, Ying Bian, Tianmin Zhen

**Affiliations:** 1 Shandong Institute of Medicine and Health Information, Shandong Academy of Medical Sciences, Jinan, Shandong Province, China; 2 Institute of Chinese Medical Sciences, University of Macau, Taipa, Macau, China; Universitat Autonoma de Barcelona, SPAIN

## Abstract

**Objective:**

To assess changes in medicine availability and prices as well as subsequent affordability during the early years of the National Essential Medicine System (NEMS) reform in China.

**Methods:**

Data were obtained from four provinces through a field survey conducted in 2010–2011. Outcome measures were percentage availability, delivery efficiency, ratios of local prices to international reference prices (MPRs), and number of days’ household income needed to purchase medicines. Prices were adjusted for inflation/deflation and purchasing power parity.

**Results:**

Under NEMS, the median MPR for essential medicines decreased from 3.27 times to 1.59 times from 2009 to 2010. The median medicine expenditure under standard treatments in 2010 equaled 1.06 days household income at a low-income level and 0.25 days household income at a middle-income level. A 25.67% reduction was observed in the average number of medicines stocked by primary healthcare facilities in 2011 compared with 2009 and the availability of essential medicines was 66.83%. During 2009–2011, suppliers could respond to 98.24% of the purchasing orders raised by primary healthcare facilities, and 89.32% of the order amounts could be delivered.

**Conclusions:**

The market prices of essential medicines greatly decreased in China after the establishment of NEMS and showed improved affordability in the short term. However, current medicine prices remain high compared to international reference prices. Medicines were often unaffordable for economically backward residents. Future policies still need to target medicine availability as well as affordability.

## Introduction

The concept of essential medicines was proposed by the World Health Organization (WHO) in 1975 and was heralded as a major breakthrough in health care. Its original intention was to ensure access, quality and rational use of medicines. Essential medicine has been considered the most cost-effective element of public health after immunization and key health promotion habits [1, 2]. China embraced the concept of essential medicines in 1979 and published the first edition of a national list in 1982. Although the essential medicine list has long existed in China, there has been no comprehensive national essential medicine policy until recently. The lack of an institutional basis, the failure of the government to clearly explain the detailed implementation measures for production, distribution, and reimbursement, and the need to sell medicines for profit to finance health facilities meant that essential medicines did not reach their full potential in China [3].

At this time, the essential medicine list mainly served as a base for the selection of medicine reimbursement list for social health insurance [4]. The role of essential medicines was weakened with the application of this drug reimbursement list. Drugs were more often categorized as Category A (reimbursable) and Category B (non-reimbursable) rather than essential and non-essential [5]. The fact that hospitals received profits from reselling medicines was likely the strongest historical barrier for the performance of essential medicines. In China, the government set prices for basic health care below cost to keep health care affordable and allowed a 15% profit margin on drugs to allow health facilities to survive financially [6]. However, such a pricing approach induced serious health hazards and physicians tended to over-prescribe, especially the more expensive or profitable medicines [7]. Selling non-essential drugs was obviously more profitable than essential medicines. Hence, the essential medicines were virtually non-existent. Consequently, manufacturers and distributors were reluctant to supply essential medicines that were neither preferred nor profitable [8]. Furthermore, during this period independently developed clinical guidelines were not available, especially on the utilization and medical management of essential medicines. Education on the use of new drugs was primarily provided by representatives of pharmaceutical distributors and manufacturers. It was easy to promote and reinforce the widespread perception that generic drugs were not as safe, effective, or reliable as brand drugs.

Along with the unsuccessful attempt to establish an essential medicine system in China, pharmaceutical expenditures have been growing by 15% per year, well above the rate of economic growth. This represents almost 50% of total health spending in China, compared with 18% in countries of the Organization for Economic Co-operation and Development [9, 10]. This proportion is also above the average of most developing countries [11]. WHO statistics showed drug expenditure in developing countries accounted for 24% to 66% of total expenditure on health. Based on the Chinese Health Statistical Digest, Chinese households devoted about 40% to 60% of their out-of-pocket healthcare expenditures to medicines between 1995 and 2008 [12]. Problems in the pharmaceutical sector have become a major source of public criticism. How to improve access to medicines has been one of the important objectives of Chinese healthcare policy-making.

In April 2009, the Chinese government released a new healthcare reform and proposed the establishment of the National Essential Medicine System (NEMS) and regarded it as one of the five top priorities in this three-year plan (2009–2012) [13]. In its initial implementation, the NEMS targeted primary healthcare facilities (PHCs) with policies designed to increase the availability of essential medicines and reduce patients’ economic burden on purchasing medicines. The NEMS policies covered essential medicines’ selection, production, procurement, distribution, pricing, utilization and reimbursement. The core was the essential medicine list, which was decided at the national level and could be supplemented at the provincial level. The latest version of the national essential medicine list (NEML) includes 520 medicines (317 western medicines and 203 Chinese traditional medicines). Another key policy element was a zero mark-up policy, under which essential medicines were sold to patients at cost and there was no profit to the hospital for the sale.

Furthermore, all essential medicines should be procured from a province-wide centralized bidding system, which aims to minimize distribution intermediaries and costs. The tender process invited pharmaceutical manufacturers and distributors to bid for “price and volume” and “economic and technical case” (Two Envelope Tendering System). The outcome of the tender process is an agreed price struck with suppliers, and medicines are therefore procured for the whole province at agreed prices and distributed to PHCs subject to these collective purchasing arrangements. PHCs must obtain their medicines through suppliers appointed to a panel arising from the tender outcomes. PHCs customarily raise a monthly purchasing order (including supplier-specific types and volumes of medicines) and submit to their regional system through a county platform. The regional system then invites expression of interests from distributors for each type of medicines. Suppliers finally select their preferred distributors (or themselves) to distribute medicines [14].

By July 2011, all 31 provinces had adopted the NEML and medicine zero-profit policy in public PHCs and established a province-based centralized procurement system [15]. Currently, considerable research has been done on evaluating the impact of NEMS. Most present studies focused on the program’s impact on medicine prices and reported that the implementation of NEMS reduced medicine prices [16–19]. However, few studies were properly designed to examine whether Chinese residents could afford the reduced prices. Additionally, very few studies have focused on medicine availability. In many countries, both pricing and availability are likely to be key factors that influence access to medicines across a range of medical conditions [20]. The objective of this research is to assess changes in medicine availability and prices as well as subsequent affordability during the early years of the NEMS reform in China.

## Methods

### Research design

A Before versus After Comparison was designed with two time periods of before and after NEMS. There were mainly considered for the following reasons. During the NEMS reform, the PHCs in the same county implemented programs nearly at the same time and it was impossible to split them into control and experimental groups. As for comparisons between counties with and without programs, it was easy to attribute differences in their conditions to differences in the implementation of NEMS, thereby influencing the judgment. Therefore, we finally conducted a comparative study of pre- and postreform periods within a county.

### Ethics statement

Our research had no direct contact with human subjects. The study protocol was reviewed and approved by the Ethics Committee of the Institution of Medicine and Health Information, Shandong Academy of Medical Sciences.

### Sampling and data collection

The four Chinese provinces, Shandong, Zhejiang, Anhui and Ningxia, were chosen as research areas, representing different levels of socioeconomic status (SES). Shandong and Zhejiang are located in the eastern part of China, representing the developed regions of China. Anhui is part of central China and is an example of a moderately developed region. Ningxia is an undeveloped area in northwest China. All four provinces implemented NEMS from 2010. Anhui began on January 1, 2010 and Zhejiang on February 25, 2010. Shandong began on March 1, 2010 and Ningxia on April 1, 2010. The sample counties in this study were the first pilot areas. All the public-owned PHCs in these pilot areas had to purchase and use essential medicines, and all the essential medicines had to be sold with zero mark-up. Therefore, the data were collected from 2009 for the prereform period and from 2010 and 2011 for the postreform period.

The survey in Zhejiang and Anhui was conducted in 2010. It was based on the program “Mid-term Evaluation on Implementation Effects of the National Essential Medicine System,” organized by the Chinese National Development Reform Committee. Two cities in each selected province were chosen by stratified sampling (one with high SES, the other with low). From each of the four selected cities three counties were randomly chosen. All state-owned PHCs in the 12 chosen counties were investigated; the total number was 113.

The survey in Shandong and Ningxia was undertaken in 2011. A county with a medium size and middle development level in Shandong was selected for sampling. All PHCs in this county were investigated; the total number was 17. In addition, a total of 16 PHCs in Ningxia were selected through stratified sampling. All counties were already divided by Ningxia local officials into three tiers of SES: high, middle and low. One county was randomly selected from each of the three SES strata. Based on the size of the county, 4–8 PHCs were randomly selected from each of the selected counties.

In total, one hundred and forty-six public PHCs were included in this survey in these four provinces. Their distribution is shown in Table 1.

**Table 1:** The quantity and distribution of PHCs in the field survey.

Study setting	No. of PHCs
Anhui	26
Zhejiang	87
Shandong	17
Ningxia	16
Total	146

During data collection, two separate questionnaires were used to collect the price information and inventory information of essential medicines in the information division of PHC. Drug-price questionnaire items included drug name, dosage, form, price, and sales. At each selected PHC, only drugs used in August 2009 and August 2010 with the same strengths and dosage forms were included in the price questionnaire survey. And their information for August 2009 and August 2010 were collected. Questions on drug-inventory questionnaire addressed the topics of procurement and distribution, including the number of medicines stored at PHC, the number of orders raised by PHC in a year, the number of orders delivered by distributors, the amount of essential medicines ordered by PHC in a year, and the amount of essential medicines actually received.

The questionnaire survey on medicine prices was undertaken in four provinces and the questionnaire survey on medicine inventory was undertaken in Shandong and Ningxia. All information from 2009, 2010, and 2011 was obtained through the drug information management system at PHC. The completed forms were to be checked on a daily basis. A follow-up telephone call was placed if there was any important information missing.

### Data analysis

#### Method used for determining price changes

The price of medicines was expressed as median price ratio (MPR), which was the ratio of a medicine’s median unit price across outlets to the international reference price (IRP) [21]. The IRP was sourced from the Management Sciences for Health (MSH) published in 2009 [22]. Price data has been corrected for inflation or deflation between the survey year and the base year (2009) using the consumer price index (CPI), and also adjusted for the purchasing power parity (PPP) of the national currency [23–24]. Changes in MPR values not only indicated the price changes of essential medicines, but also reflected the price level compared with the international level. Normally, an MPR of 1 or less indicates an efficient public-sector procurement system.

To allow comparisons and ensure representativeness, a short “core” list of medicines was selected as a basis for analysis. This refers to medicines included in both the WHO Global Core List/Western Pacific Regional Core List [25] and China NEML. Only these medicines were included in the price comparison from the survey medicines. During analysis, the price of a drug was evaluated only when data had been obtained from four or more PHCs. All compared drugs had the same generic name but were not necessarily from the same producers. Drugs (same generic name) with different strengths were considered as separate drugs. For example, enalapril tablet 10 mg and enalapril tablet 5 mg were considered as two medicines. In total, twenty medicines were analyzed in this study.

#### Measurement of medicine affordability

According to the methodology developed by the WHO and Health Action International, affordability was estimated by the number of days’ income needed to purchase courses of treatment for common conditions [26]. Treatment costs refer to medicines only and exclude the additional costs of consultation and diagnostic tests. The cost of a medicine was calculated by its daily dose, course of treatment, and price. The course of treatment was 7 days for acute conditions and 30 days for chronic diseases. Daily income was considered using the national relative poverty line, which was 1196 yuan/year (3.3 yuan/day) and the per capita net income of rural households, which was 5153 yuan/year (14.3 yuan/day). These can represent the income levels for the low-income population (LIP) and middle-income population (MIP) in China, respectively. Income figures were retrieved from the report of the Chinese Ministry of Human Resources and Social Security [27]. Basically, treatments costing one day’s income or less are considered affordable [28].

#### Measurement of medicine availability

The availability of essential medicines was a percentage referred to as the number of essential medicines stocked at each individual PHC in a year divided by the total number of medicines in the essential medicine list. This was a facility-based indicator.

#### Measurement of medicine delivery efficiency

The NEMS aims to improve availability and affordability in primary care facilities. Poor distribution of medicines to primary care facilities would affect the availability of medicines. The delivery efficiency of essential medicines was considered by the response rate of medicine delivery (RR) and the arrival rate of essential medicines (AR). The two indicators measure the resistance by industry to the NEMS and are related to the distribution of essential medicines.

RR was determined by dividing the number of orders actually delivered by distributors by the number of orders raised by PHCs in a year. AR was determined by dividing the amount of essential medicines actually received at PHCs (Yuan) by the amount of essential medicines they ordered (Yuan) in a year.

## Results

### Changes in medicine prices

Prior to the NEMS implementation, the median MPR of the twenty observed essential medicines was 3.27 times the IRPs. Of those, the MPRs of six medicines were 10 times more than their IRPs while diclofenac was the highest at 69.77. Only two medicines, captopril (0.60) and nifedipine (0.44), were at lower prices than the IRPs.

After NEMS was implemented, all MPRs declined except for omeprazole. Significantly, the MPRs of the four medicines were reduced by more than 10, including diclofenac, simvastatin, enalapril and ibuprofen (600 mg). They were the four most expensive drugs in 2009. The median MPR of the twenty medicines was 1.59 times the corresponding IRP after NEMS. Eight medicines were at lower prices than the IRPs. The prices of 12 drugs were at the international level. However, some drugs still cost more than ten times the international reference prices, including omeprazole (10.09), ibuprofen (30.05), diclofenac (39.51), simvastatin (19.94), enalapril 10 mg (26.14) and enalapril 5 mg (14.61). Table 2 presents the detailed results of the MPR changes. Table 3 shows the province-specific results; it shows that the same downward trend was observed in the MPRs in the four provinces in response to NEMS, although the amounts of the decreases were different in the different provinces.

**Table 2:** The median price ratios for essential medicines before and after NEMS.

Generic name	Strength	IRP (yuan)	No. of PHCs	Before		After	
				Median unit price (yuan)	MPR	Median unit price (yuan)	MPR
Albendazole	200 mg	0.09	23	1.25	13.84	0.91	9.76
Amoxicillin	250 mg	0.09	62	0.29	3.32	0.15	1.65
Omeprazole	20 mg	0.04	62	0.44	10.00	0.46	10.09
Valproic acid	200 mg	0.06	4	0.13	2.13	0.11	1.80
Ibuprofen	600 mg	0.04	10	1.95	45.30	1.34	30.05
Ibuprofen	200 mg	0.04	33	0.06	1.58	0.03	0.76
Diazepam	5 mg	0.02	19	0.11	6.64	0.04	2.47
Metformin	500 mg	0.04	65	0.11	2.95	0.06	1.54
Ciprofloxacin	250 mg	0.10	33	0.33	3.22	0.10	0.96
Metronidazole	200–250 mg	0.03	66	0.05	1.98	0.03	1.18
Captopril	25 mg	0.07	63	0.04	0.60	0.03	0.37
Ranitidine	150 mg	0.09	57	0.13	1.51	0.06	0.65
Hydrochlorothiazide	25 mg	0.01	39	0.03	2.88	0.01	0.86
Diclofenac Sodium	50 mg	0.02	24	1.33	69.77	0.78	39.51
Cephalexin	250 mg	0.23	41	0.28	1.25	0.15	0.66
Ceftriaxone	1 g/vial	1.80	65	10.00	5.55	1.73	0.93
Nifedipine	10 mg	0.07	38	0.03	0.44	0.01	0.16
Simvastatin	20 mg	0.12	39	4.66	38.59	2.49	19.94
Enalapril	10 mg	0.04	18	1.71	47.78	0.97	26.14
Enalapril	5 mg	0.05	13	0.80	17.84	0.68	14.61
**Median**					3.27		1.59
**[25%, 75%]**					[1.88, 14.84]		[0.84, 11.22]

Note: United States dollar = 6.8 Chinese yuan

**Table 3:** The median price ratios for essential medicines before and after NEMS in four provinces.

Region	Median MPR	
	Before	After
Zhejiang	4.55	2.05
Anhui	2.26	1.42
Shandong	2.07	1.07
Ningxia	1.07	0.75

### The affordability of medicines

Table 4 shows the affordability of drugs before and after NEMS. At the median, medicines for standard treatment cost 0.51 days’ income of MIP and 2.18 days’ income of LIP in 2009. By 2010, the affordability of most medicines had been reasonable (with standard treatment costing a day’s income or less) for MIP, except for ibuprofen (1.31 days’ income), diclofenac (3.25 days’ income), simvastatin (5.21 days’ income), and enalapril (2.03–2.83 days’ incomes). However, only nine medicines cost below a day’s income of LIP. The median medicine expenditure under standard treatments in 2010 equaled 1.06 days household income at a low-income level and 0.25 days household income at a middle-income level. Diclofenac, simvastatin and enalapril were the most expensive for both groups.

**Table 4:** The affordability of medicines before and after NEMS.

Generic name	Strength	Median price (yuan)		Defined daily dose	Treatment course (day)	Days’ income for MIP		Days’ income for LIP	
		Before	After			Before	After	Before	After
Albendazole	200 mg	1.25	0.91	2	7	1.22	0.89	5.27	3.83
Amoxicillin	250 mg	0.29	0.15	3	7	0.42	0.22	1.83	0.94
Omeprazole	20 mg	0.44	0.46	1	30	0.93	0.97	4	4.17
Valproic acid	200 mg	0.13	0.11	3	30	0.82	0.71	3.52	3.06
Ibuprofen	600 mg	1.95	1.34	2	7	1.91	1.31	8.22	5.63
Ibuprofen	200 mg	0.06	0.03	4	7	0.12	0.06	0.51	0.25
Diazepam	5 mg	0.11	0.04	1	30	0.23	0.09	1.01	0.39
Metformin	500 mg	0.11	0.06	2	30	0.45	0.24	1.95	1.05
Ciprofloxacin	250 mg	0.33	0.10	2	7	0.32	0.1	1.37	0.42
Metronidazole	200–250 mg	0.05	0.03	6	7	0.15	0.09	0.63	0.39
Captopril	25 mg	0.04	0.03	2	30	0.17	0.11	0.72	0.47
Ranitidine	150 mg	0.13	0.06	2	30	0.56	0.25	2.4	1.07
Hydrochlorothiazide	25 mg	0.03	0.01	1	30	0.07	0.02	0.31	0.1
Diclofenac Sodium	50 mg	1.33	0.78	2	30	5.55	3.25	23.93	14
Cephalexin	250 mg	0.28	0.15	3	7	0.41	0.23	1.77	0.97
Ceftriaxone	1 g/vial	10.00	1.73	1	7	4.89	0.85	21.07	3.65
Nifedipine	10 mg	0.03	0.01	3	30	0.21	0.08	0.89	0.33
Simvastatin	20 mg	4.66	2.49	1	30	9.76	5.21	42.05	22.45
Enalapril	10 mg	1.71	0.97	1	30	3.59	2.03	15.48	8.75
Enalapril	5 mg	0.80	0.68	2	30	3.35	2.83	14.41	12.19
Median						0.51	0.25	2.18	1.06
[25%, 75%]						[0.23, 2.27]	[0.10, 1.06]	[0.98, 9.77]	[0.41, 4.54]

Note: United States dollar = 6.8 Chinese yuan

### The availability of essential medicines

**Fig 1:**
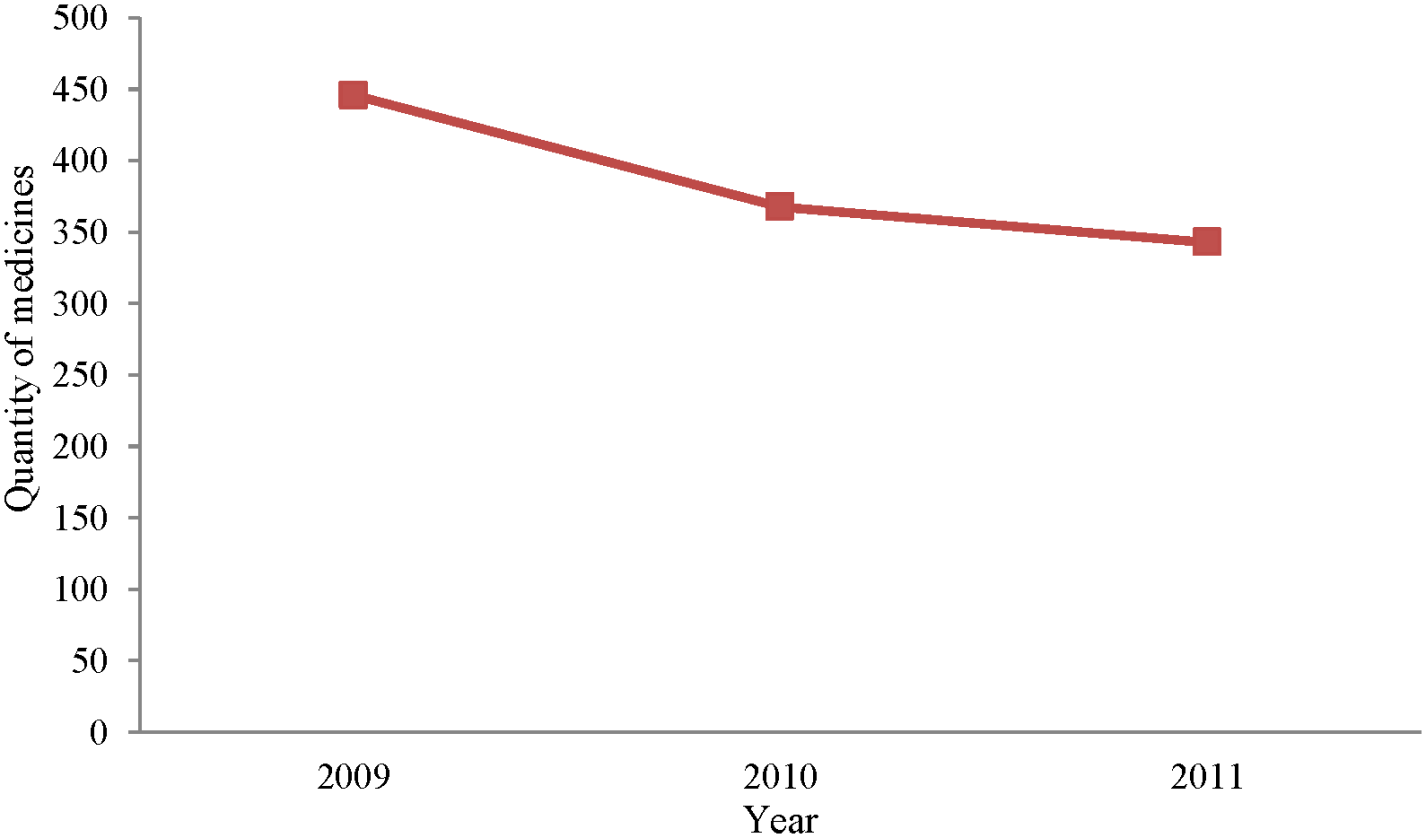
The quantity of medicines stocked by primary healthcare centers, 2009–2011.

The amount of medicines stored by PHCs decreased with the implementation of NEMS, as shown in Fig 1. The average number of medicines stocked was 343 in 2011, a 25.67% reduction compared with 2009. However, the proportion of essential medicines increased significantly. Table 5 lists the availability of essential medicines in PHCs from 2009 to 2011. The availability of national essential drugs increased in 2011 compared to 2009, but the overall availability of essential medicines did not appear to increase. A total of 66.83 percent essential medicines could be available in PHCs by 2011.

**Table 5:** The availability of essential medicines in PHCs from 2009 to 2011.

	Availability of national essential drugs, %			Availability of provincial essential drugs, %			Availability of essential medicines, %		
	Mean	SD	95% CI	Mean	SD	95% CI	Mean	SD	95% CI
2009	61.95	24.20	44.64–79.27	75.35	38.21	45.98–104.72	68.30	31.45	53.14–84.46
2010	69.22	19.43	61.54–76.91	61.77	21.89	52.93–70.61	65.57	20.81	59.83–71.30
2011	72.58	20.26	64.56–80.59	61.09	19.36	53.43–68.75	66.83	20.46	61.25–72.42

Note: *P* value refers to one-way ANOVA for trend

### The efficiency of medicine delivery

Table 6 shows changes in the efficiency of medicine delivery between 2009 and 2011. The efficiency of medicine delivery was relatively stable, although a slight declining trend was observed in the means of AR and RR during these three years. Under NEMS, suppliers could respond to 98.24% of the purchasing orders raised by PHCs, and 89.32% of the order amounts could be delivered, on average.

**Table 6:** The efficiency of medicine delivery from 2009 to 2011.

	Response rate of delivery, %			Arrival rate of essential medicines, %		
	Mean	SD	Median [25%, 75%]	Mean	SD	Median [25%, 75%]
2009	99.68	1.63	100 [100, 100]	91.66	21.95	100 [97.42, 100]
2010	97.67	11.49	100 [100, 100]	90.33	21.41	99.12 [91.11, 100]
2011	97.56	10.28	100 [100, 100]	86.45	27.49	99.27 [89.69, 100]
Total	98.24	9.10	100 [100, 100]	89.32	23.65	99.59 [91.43, 100]

## Discussion

### Medicine prices

One of the most important goals of NEMS is to lower drug prices. The results of this study support the positive effect of NEMS on price control in the short term. This finding has also been confirmed by many studies. The average price decline reported in the literature is 20% [5, 16, 29]. Previous studies have suggested that centralized procurement and the zero mark-up policy are the major contributors to the price reduction [5, 11, 18]. They are the two key elements of the Chinese NEMS. Before reform, procurement occurred at facility levels; PHCs procured drugs directly from suppliers at various prices. Following implementation of NEMS, each province established unified bidding, purchasing, and supply procedures for drugs sold in PHCs. Centralized procurement improved the efficiency of medicine delivery in general. These efficiencies combined with higher volume purchasing have resulted in reductions in medicine prices [30]. The government reported that the tender price dropped by 33% on average compared to the last regional procurement, and declined by an average of 55% compared to the national retail reference price [31]. Furthermore, the zero mark-up policy cancels the profit margins on essential medicines in PHCs, which also represents a reduction in retail prices.

The province-specific results showed there were differences in the price reductions in the four provinces. It may be related to the implementation of regional bidding procurement policies, because the central government did not include any specific policy on procurement and logistics [14]. In addition, since 2006, Ningxia has implemented the “trinity” reform of drug tender policy (unified procurement, unified distribution and unified price), which played an important role in price control [32]. The overall price level was relatively reasonable and therefore the price change in Ningxia was not as obvious as the other three provinces after NEMS.

Furthermore, it is noteworthy that the current MPR of some medicines observed in our study was still greater than 1. Therefore, it can be concluded that the efficiency of the public-sector procurement system has yet to be improved, and the price of drugs still has room to fall. Therefore, there is a need for a system that uses routine data to monitor standardized indicators of medicine prices. It is also important to explore the establishment of a drug price warning mechanism based on international reference prices.

Price control is an important target of NEMS, but it does not mean the lower the price the better. The excessive pursuit of lower prices in competitive bidding may increase the following risks: a) shortages of certain specific medicines—the bidding prices would severely curtail the profit of some manufacturers and distributors (“the winning death”) [33]; b) potential quality risks—manufacturers may produce substandard medicines to ensure their profits. In this investigation, the MPR of nifedipine and captopril was 0.44 and 0.60, respectively, and lower than international reference prices before NEMS. Following implementation of the program, their prices were further reduced. Although it is beneficial to patients, there could be the risks mentioned above. It would add a greater burden to patients if these good cost-effective medicines disappeared from the market.

Given the anticipated intensive competition, a well-regulated bidding procurement system is required. The quality and safety of the medicines should be determined before the commercial tender evaluation. During commercial bidding, an in-depth comprehensive assessment should be conducted for significantly lower prices to avoid vicious competition. Regarding these very cheap generic medicines, two envelope open tenders, one with the technical parameters and a second containing the price quotes, can be replaced by one envelope, which is only for technological specifications [19].

### Medicine affordability

The analysis of affordability shows the price levels of essential medicines associated with individual purchasing power. The affordability of essential medicines has improved in China after the implementation of NEMS. By 2010, the medicines had reasonable affordability for most MIP, with standard treatment costing 0.25 days of users’ incomes. However, medicines were still unaffordable for LIP, with standard treatment costing 1.06 day’s income. Additionally, it is important to note that individuals or families who need multiple medications might face unaffordable drug costs even if individual therapy seems affordable. One example is provided below of a family where the father has an ulcer and his child has an infection, treated with omeprazole and ceftriaxone, respectively. If the household income is equivalent to the middle-income group, their medical expenses will be 1.82 days’ income.

By the end of 2010, 96 percent of China’s rural residents participated in the new rural cooperative medical care system, but the level of protection was not high. In 2010, the total medical expense for rural outpatients was 47.5 Yuan. The drug cost was 28.7 Yuan, accounting for 60.4% of the total expense. Furthermore, the medical expense per capita for hospitalized patients was 1004.6 Yuan. The drug cost was 531.1 Yuan, accounting for 52.9% of the total expense [34]. Therefore, in addition to further price control, cost-sharing of social health insurance should be fully activated to ensure access to medicines, such as establishing a special subsidy mechanism for some serious diseases, and implementing a medical voucher plan for low-income populations.

### Medicine availability

Our research showed that the number of medicines stored by PHCs decreased from 2009 through 2011. This may be because drugs not included in the list are not allowed to be used in PHCs. The result of our study showed that the average number of medicines stocked by PHCs was 343 in 2011. A similar quantity was indicated in another study, involving 431 PHCs across China. According to their findings, the average number of drugs stocked by PHCs by the end of 2010 was 322 [35]. Based on our results, more than 60% of medicines in the essential medicine list had been available in PHCs by 2011. A survey in 2007 showed PHCs in Shandong province supplied a median of 26% of western medicines and 25% of Chinese traditional medicines on the NEML and these figures in Gansu were 19% and 33%, respectively [8]. A comparison between 2007 and 2011 showed that the availability of essential medicines improved following the implementation of NEMS. Furthermore, this result was higher than the median value in over 40 mainly low- and middle-income countries (44%) in 2011[36].

The ability of primary care facilities to provide adequate health care is directly related to the availability of medicines. It is necessary to improve distribution efficiency and ensure the supply of essential medicines. According to research in Fujian province, China, 42 products on the essential medicine list could not be easily procured. The main reasons included lack of a supplier, sole source manufacturers, and firms’ non–acceptance of the tendering price [30]. The problems in drug supply might not only compromise service quality but also lead to a misperception among patients that doctors are not willing to provide care that was not profitable [37]. Many innovative ways could be explored. For example, medicines can be delivered with the aid of logistics services (such as the postal service) that can provide wide coverage in China. DHL has partnered with UNICEF Kenya to improve the medical supply chain [38].

### Limitations

The present study has four limitations that need to be acknowledged. First, due to lack of matched control sites, the findings could not be persuasive with a pre-post study design. However, the NEMS is the only reform measure related to the transformation of the pharmaceutical sector in the context of new Chinese healthcare reform, so the observed changes in drug prices and affordability and availability are probably attributable to the NEMS. Second, the results of this study were based on four provinces. They were selected to represent different socioeconomic development levels but were not random. The conclusions of this study should be generalized to China with caution. Furthermore, since Zhejiang represented over half of the sample, the obvious decreases in MPRs compared with the other three provinces might overstate the positive effect of the NEMS on price control. Third, to make the survey manageable and to enable comparability, the changes in medicine prices as well as subsequent affordability were determined based on a specific list of survey medicines. The reliability of some results might be skewed because of the limited data. Fourth, the present study was conducted shortly after the introduction of the NEMS in China. The effectiveness and impact of the NEMS may not be particularly significant. Despite the limitations of the present study, the main changes observed following the implementation of the NEMS were predictable. The findings match those of other evaluations of the NEMS and the province-specific results are consistent in some respects.

## Conclusions

To conclude, the market prices of essential medicines greatly decreased in China after the establishment of the NEMS and showed improved affordability in the short run. However, current medicine prices remained high compared to international reference prices. Medicines were often unaffordable for economically backward residents. Future policies still need to target medicine availability as well as affordability.

## Supporting information

S1 File Study questionnaire. (XLSX)

S2 File Data set. (XLSX)
